# Zinc Limitation Induces a Hyper-Adherent Goliath Phenotype in *Candida albicans*

**DOI:** 10.3389/fmicb.2017.02238

**Published:** 2017-11-14

**Authors:** Dhara Malavia, Laura E. Lehtovirta-Morley, Omran Alamir, Elisabeth Weiß, Neil A. R. Gow, Bernhard Hube, Duncan Wilson

**Affiliations:** ^1^Medical Research Council Centre for Medical Mycology at the University of Aberdeen, Aberdeen Fungal Group, Aberdeen, United Kingdom; ^2^School of Biological Sciences, University of East Anglia, Norwich, United Kingdom; ^3^Department of Microbial Pathogenicity Mechanisms, Leibniz Institute for Natural Product Research and Infection Biology – Hans Knöll Institute, Jena, Germany; ^4^Center for Sepsis Control and Care, University Hospital, Jena, Germany; ^5^Institute of Microbiology, Microbial Pathogenicity, Friedrich Schiller University, Jena, Germany

**Keywords:** *Candida albicans*, fungal pathogen, morphology, morphogenesis, nutritional immunity, micronutrients, zinc, adhesion

## Abstract

Pathogenic microorganisms often face acute micronutrient limitation during infection due to the action of host-mediated nutritional immunity. The human fungal pathogen *Candida albicans* is polymorphic and its morphological plasticity is one of its most widely recognized pathogenicity attributes. Here we investigated the effect of zinc, iron, manganese, and copper limitation on *C. albicans* morphology. Restriction of zinc specifically resulted in the formation of enlarged, spherical yeasts, a phenotype which we term Goliath cells. This cellular response to zinc restriction was conserved in *C. albicans*, *C. dubliniensis* and *C. tropicalis*, but not in *C. parapsilosis*, *C. lusitaniae* or *Debaryomyces hansenii*, suggesting that it may have emerged in the last common ancestor of these related pathogenic species. Cell wall analysis revealed proportionally more chitin exposure on the Goliath cell surface. Importantly, these cells were hyper-adherent, suggesting a possible role in pathogenicity. Interestingly, the zincophore-encoding gene *PRA1* was expressed by Goliath cells in zinc limited media and lack of Pra1 inhibited both cellular enlargement and adhesion. Goliath cells represent a further layer of *Candida* phenotypic plasticity.

## Introduction

In order to prevent the growth of invading microbes and restrict the progression of infection, the mammalian immune system has developed sophisticated mechanisms to actively withhold certain essential trace nutrients such as iron and zinc. This process is called nutritional immunity (Cassat and Skaar, 2012; Hood and Skaar, 2012). Therefore, in order for a pathogen to survive this nutritionally restrictive environment within its infected host, it must have evolved strategies to overcome nutritional immunity.

Transition metals are essential to life as approximately a third of all proteins interact with a metal cofactor (Waldron et al., 2009; Hood and Skaar, 2012) and within the context of nutritional immunity, the host and invading microbe battle for these micronutrients resulting in a ‘nutritional tug-of-war’ (Anzaldi and Skaar, 2010; Skaar, 2010). The importance of host-driven iron sequestration to control microbial infections is well established (Skaar, 2010). More recently, an emerging role for zinc in nutritional immunity has been recognized (Kehl-Fie and Skaar, 2010). Zinc is an essential trace metal crucial for various biological processes and 9% of eukaryotic proteins are predicted to be zinc metalloproteins (Andreini et al., 2009). Furthermore, eukaryotic cells rely on a significant number of zinc dependent transcription factors for gene regulation (Andreini et al., 2006; Hood and Skaar, 2012). Moreno et al. (2007) demonstrated the importance of zinc acquisition in pathogenicity of *Aspergillus fumigatus*. Similarly, deletion of zinc transporters in *Cryptococcus gattii*, *Cryptococcus neoformans*, and *Histoplasma capsulatum* has been shown to attenuate virulence in murine models of infection; thus providing evidence that the response of fungal pathogens to zinc limitation is important for virulence (Amich et al., 2014; Jung, 2015; Schneider et al., 2015; Dade et al., 2016; Do et al., 2016).

Polymorphism in several fungal pathogens is a well-recognized virulence factor (Boyce and Andrianopoulos, 2015). *H. capsulatum*, *Blastomyces dermatitidis*, *Paracoccidioides brasiliensis*, *Talaromyces marneffei* and most other pleomorphic fungal pathogens grow as filamentous forms in the environment, but transition to pathogenic yeast phases insides their infected hosts (Kane, 1984; Maresca and Kobayashi, 1989). *C. neoformans* also transitions to pathogenic yeast growth during infection and Okagaki et al. (2010), Zaragoza et al. (2010), Okagaki and Nielsen (2012) have demonstrated the formation of giant Titan cells, which were resistant to phagocytosis by macrophage-like cells.

*Candida albicans* is a polymorphic fungus and its morphological plasticity is recognized as a key virulence attribute (Liu, 2001; Sudbery et al., 2004; Whiteway and Bachewich, 2007). During infections, filamentous forms of *C. albicans* are known to penetrate epithelial and endothelial cells and mucosal barriers causing damage to host tissue (Sudbery, 2011; Tyc et al., 2014). Multiple studies have shown that morphological transitions play an important role in host–pathogen interactions for this fungus. However, the physiological response of *C. albicans* to nutritional immunity is poorly understood. *C. albicans*, although a member of the human gut microbiota, is frequently responsible for superficial as well as life threatening disseminated infections. Kullberg and Arendrup (2015) reported more than 250,000 people worldwide suffer from severe invasive Candidiasis each year with high mortality rates (Kullberg and Arendrup, 2015).

Here we describe the physiological response of *C. albicans* to zinc starvation. We found that zinc (but not iron, manganese, or copper) deprivation causes *C. albicans* to transform to a giant yeast cell phenotype. Combined phylogenetic-phenotypic analysis indicates that this cellular-enlargement response to zinc limitation is species-specific, arose in a common ancestor of *C. albicans* and *C. tropicalis* and was not observed in several other tested *Candida* species. Importantly, these cells exhibit enhanced adhesion – a property normally associated with the hyphal morphology. We propose the term “Goliath” cell for this giant, hyper-adherent *C. albicans* phenotype.

## Results

### Zinc Starvation Induces Cellular Enlargement in *Candida albicans*

**FIGURE 1:**
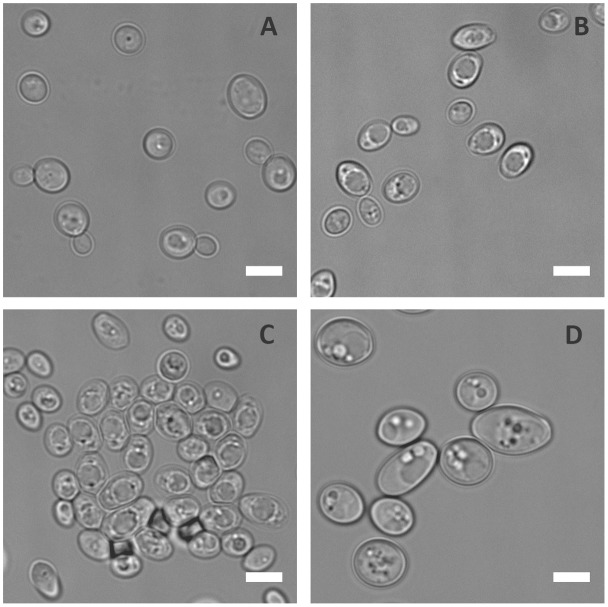
Physiological response of *C. albicans* to metal starvation. *C. albicans* (BWP17 + Clp30) cells subjected to copper **(A)**, iron **(B)**, manganese **(C)**, and zinc **(D)** starvation by incubating in limiting medium independently lacking these metals at 30°C, 200 rpm for 3 days. Experiment performed twice. DIC images show that of the metals tested only zinc starvation resulted in cellular enlargement in *C. albicans*. Scale bar represents 5 μm.

To prevent infection, the host immune system withholds certain essential nutrients like iron and zinc in a process known as nutritional immunity. In order to understand the physiological responses of *C. albicans* to metal starvation, the laboratory wild type (WT) strain (BWP17+Clp30), was subjected to iron, manganese, copper or zinc starvation *in vitro* for 3 days. Following incubation in metal limiting media, cells were observed microscopically. **Figure 1** shows that, of the metals tested, zinc starvation induced cellular enlargement in *C. albicans*.

**FIGURE 2:**
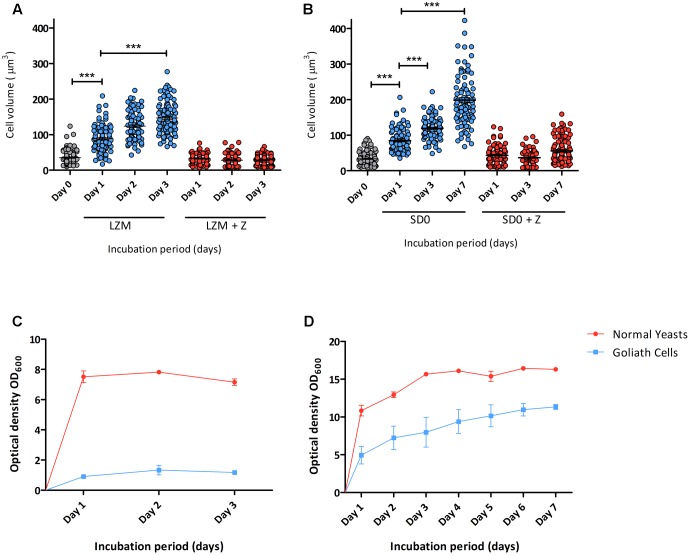
Developmental kinetics of *C. albicans* Goliath cell formation under zinc limitation. *C. albicans* cells pre-grown in SD medium were **(A)** incubated in LZM or LZM + Z over 3 days or **(B)** in SD0 or SD0 + Z over 7 days. Cells were imaged at indicated time points and axes diameters measured using ImageJ. Cell volumes were calculated by *V* = 4/3 π *ab*^2^. Each data column shows >100 cells from at least two independent experiments. Both zinc starvation media induced significant cellular enlargement (^∗∗∗^ < 0.001, Stat. test [ANOVA]). One data point (530 μm^3^) is omitted from SD0 Day 7 **(B)**. Optical density (OD_600_) was determined for cultures incubated in **(C)** LZM (±zinc) every 24 h for 3 days and **(D)** SDw/oZ (±zinc) every 24 h for 7 days. Data are from three independent experiments. Error bars represent the standard error of the mean.

Next, the kinetics of zinc limitation-induced cellular gigantism was assessed. *C. albicans* cells were incubated in limited zinc medium (LZM) and in medium containing zinc (LZM + Z). Cells were analysed microscopically daily for 3 days and cell volume determined. **Figure 2A** shows that significant cellular enlargement was observed as early as day 1 of zinc starvation, and an average cell volume of 146 μm^3^ (±43.6 μm^3^) was reached by day 3. This is in contrast to regular yeast cells which exhibit average cell volumes of 28–35 μm^3^. To confirm this was not a medium-specific response, *C. albicans* was incubated in another synthetic defined medium lacking zinc (YNB-zinc drop out – “SD0”). Again a similar cellular enlargement was observed in SD0 with cells reaching an average volume of 119 μm^3^ by day 3 and 198 μm^3^ by day 7 (**Figure 2B**). **Figures 2C,D** show that *C. albicans* growth was inhibited in a zinc-dependent manner in these experiments. To ensure that OD_600_ measurements did not represent dead cells, colony forming units (cfu) were determined. Yeast cells inoculated into LZM to a cell density of 3 × 10^6^ cfu/ml on day 0 increased by day 1 to 1 × 10^7^ cfu/ml. Viability (cfu/ml) then remained constant for up to 7 days.

Enlarged yeast cells have previously been described for *C. neoformans*, where they are known as “Titan Cells” (Zaragoza et al., 2010). Here we propose the term *C. albicans* “Goliath cells” for this cellular enlargement observed upon zinc depletion.

### Origin of Cellular Enlargement in *Candida*

**FIGURE 3:**
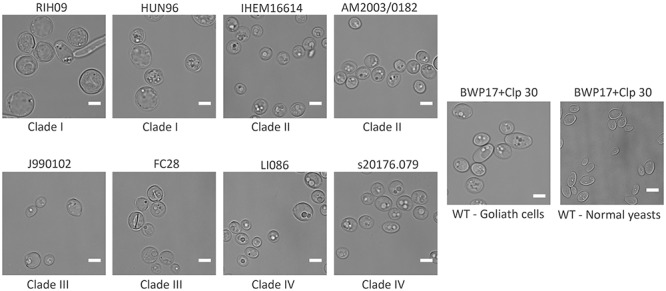
Cellular gigantism in multiple *C. albicans* clinical isolates. Indicated *C. albicans* clinical isolates pre-grown in SD medium were incubated in LZM for 3 days at 30°C, 200 rpm. DIC images show that all the tested clinical isolates can enlarge to varying degrees upon zinc depletion. Clinical isolates belonging to Clade 1 enlarged to a greater degree than other isolates. All the strains are distinctly bigger than the LZM + Z control. Scale bar represents 5 μm.

BWP17+CIp30 used in this study is a laboratory strain. To determine if the observed cellular enlargement is a conserved response, multiple *C. albicans* clinical isolates spanning the four major clades of this species (Supplementary Table S1) (MacCallum et al., 2009) were incubated in LZM for 3 days and observed microscopically for cellular enlargement. All the tested clinical isolates enlarged to varying degrees upon zinc depletion (**Figure 3**), indicating that this response is a conserved feature of *C. albicans* biology.

**FIGURE 4:**
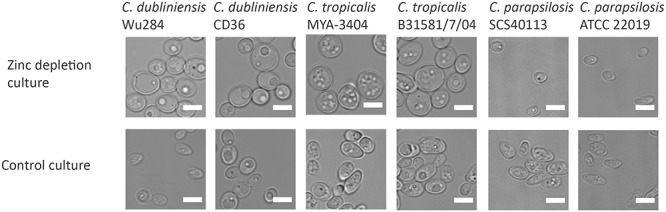
Zinc restriction-induced cellular enlargement is conserved in closely related *Candida* species. Multiple strains of *C. dubliniensis*, *C. tropicalis*, and *C. parapsilosis* were also tested for their capacity to enlarge by incubating SD-pre-grown cells in LZM (±zinc) for 3 days at 30°C, 200 rpm. All the tested strains of *C. dubliniensis* and *C. tropicalis* enlarged in response to zinc limitation, whereas *C. parapsilosis* strains did not enlarge upon zinc deprivation. Scale bar represents 5 μm.

We next questioned when during fungal evolution this property emerged. *C. albicans* is a member of the CUG-Ser clade of yeasts, which uniquely translate the CUG codon as serine instead of leucine. We therefore incubated several clinical isolates and reference strains of *C. dubliniensis, C. tropicalis, C. parapsilosis, C. lusitaniae*, and *Debaryomyces hansenii* (also known as *C. famata*) under zinc depletion and monitored their morphology. Interestingly, all the tested strains of *C. dubliniensis* and *C. tropicalis* enlarged upon zinc starvation. However, the other tested species did not exhibit the Goliath cell phenotype upon zinc deprivation (**Figure 4** and data not shown). This analysis suggests that cellular enlargement in response to zinc limitation is species-specific and may have arisen in the last common ancestor of *C. albicans*, *C. dubliniensis*, and *C. tropicalis*. All three species are pathogens of humans (Butler et al., 2009).

### The Impact of Zinc Restoration on Morphology

**FIGURE 5:**
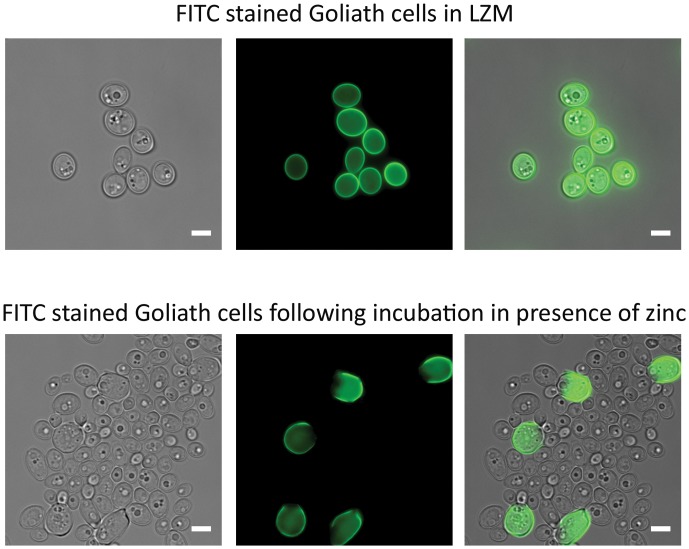
Effects of zinc restoration on Goliath cell morphology. *C. albicans* cells pre-grown in SD were incubated in LZM for 2 days to generate Goliath cells. These Goliath cells were FITC labeled (upper panel) and subsequently inoculated into SD (zinc-replete) medium to an OD_600_ = 0.5. Lower panel shows that non-labeled cells exhibit regular yeast morphology. Labeled cells remained large. Scale bar represents 5 μm.

When *C. albicans* hyphae are switched to yeast culture medium, or if the hyphal maintenance program is genetically perturbed, the hyphal compartments themselves endure and produce yeast cells (Enjalbert and Whiteway, 2005; Polke et al., 2017). We therefore investigated the fate of Goliath cells and the morphology of daughter cells produced upon zinc restoration. The Goliath cell’s surface were stained with FITC, which is maintained on the stained cell during culture, but not transferred to daughter cells. These stained cells were inoculated into SD yeast growth medium at OD_600_ = 0.5 and incubated for 24 h. Following this culture period, cell density increased to OD_600_ 10–12. Microscopic analysis revealed both large and regularly sized yeast cells in the culture, with only the large cells exhibiting FITC fluorescence (**Figure 5**). The ratio of regular-sized un-labeled to large FITC-labeled cells was 20:1 (4.7% FITC-labeled). Therefore, Goliath cells maintain their size, but generate regular-sized daughter cells in response to zinc re-supplementation.

**FIGURE 6:**
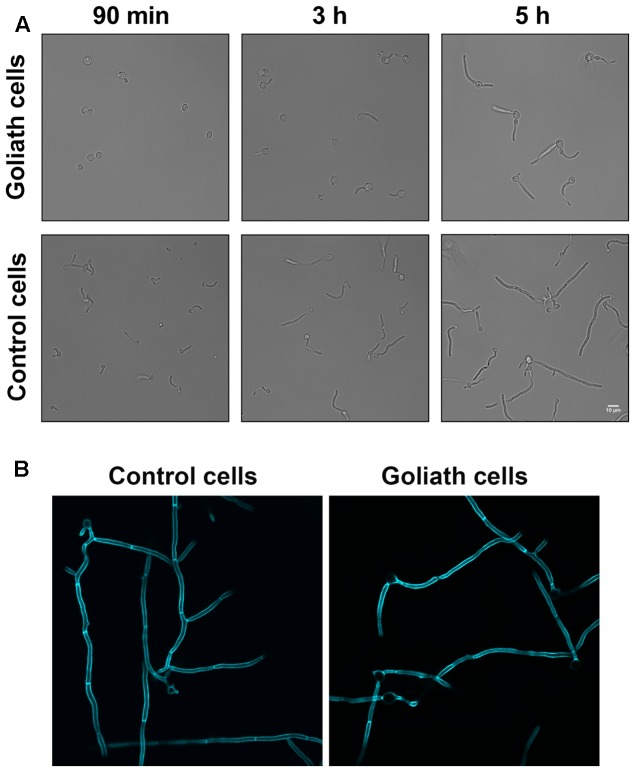
Hyphae induction. Goliath cells and normal yeast cells incubated in the presence of hyphae inducing media (RPMI) for up to 5 h **(A)** or 6 h at 37°C and stained with Calcofluor White **(B)**. Goliath cells exhibit a delay in germination and hyphae formation as compared to normal yeasts. Scale bar represents 10 μm.

We next tested whether Goliath cells can form hyphae. Goliath cells and control yeasts were inoculated into the wells of tissue culture plates containing RPMI and incubated at 37°C and 5% CO_2_ – a potent inducer of hyphae. **Figure 6** shows that, as expected, the majority of control yeasts had germinated by 90 min and these hyphae continued to extend in length at 3 and 5 h post-inoculation. In contrast, Goliath cell germination was not observed until 3 h (**Figure 6A**). To test whether these filaments were true hyphae, the cells were stained with Calcofluor White. **Figure 6B** shows the presence of true hyphae formed by Goliath cells as indicated by the position of the primary septum and parallel cell wall around septa (Sudbery et al., 2004). Therefore, Goliath cells exhibit delayed filamentation in response to a strong inducer of hyphae.

### Cellular Enlargement Increases Chitin Exposure in *C. albicans*

**FIGURE 7:**
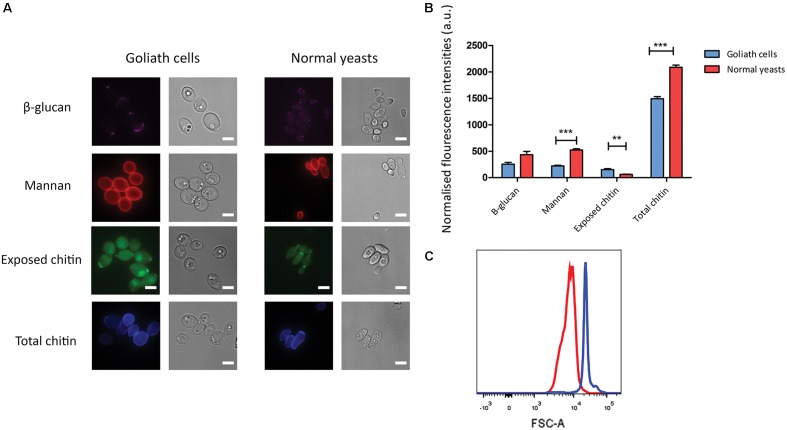
Cell wall analysis of Goliath cells. **(A)** Goliath cells stained for exposed β-glucan (Fc-Dectin), mannan (concanavalin A), exposed (WGA) and total (Calcofluor White) chitin and compared with normal yeast cells. Scale bar represents 5 μm. **(B)** Mean fluorescence intensities of respective cell wall components for Goliath cells and normal yeast cells as determined by flow cytometry represented as a bar graph. Data were obtained from three independent experiments and normalized by cell size **(C)**, error bars represent standard deviation and *p*-values were calculated by *t*-test. A significant increase in mean fluorescence intensity of WGA was observed Goliath cells as compared to normal yeast cells. (^∗^*P* ≤ 0.05, ^∗∗^*P* ≤ 0.01, ^∗∗∗^*P* ≤ 0.0001). **(C)** Increase in cell size determined using flow cytometry by measuring forward scatter (FSC) intensities of Goliath cells (blue) and normal yeast cells (red) (*n* > 10000). A shift in FCS peak shows that Goliath cells are significantly larger than normal yeast cells. Mean FCS intensities were compared to determine fold increase in cell size. Based on the FCS intensities, *C. albicans* Goliath cells exhibit a 3.3-fold increase in size as compared to normal yeasts. Note that relative to cell size, only chitin exposure is relative higher for Goliath cells vs. control yeasts.

*Candida albicans* yeast cells are known to remodel their cell wall in response to environmental changes and during morphological switches such as the yeast to hypha transition (Klis et al., 2001; Heilmann et al., 2011). We hypothesized that the cellular transformation to the Goliath phenotype may also be associated with cell wall remodeling. To investigate whether Goliath cells exhibit alterations in cell wall composition, cells were stained with dyes against the major cell wall components and analyzed by fluorescence microscopy (**Figure 7A**) and flow cytometry (**Figure 7B**). These were: concanavalin A (mannan), FC-dectin (β-glucan), Calcofluor White (total chitin) and FITC-conjugated wheat-germ agglutinin (exposed chitin).

Flow cytometry measurements indicated increased mannan (1.4-fold), β-glucan (2-fold) and total chitin (3.8-fold) levels in Goliath cell walls compared to control yeast cells. However, the defining feature of Goliath cells is their size. In agreement with our microscopic measurements (**Figure 2A**), forward light scatter flow cytometry measurements (a proxy for particle size) of Goliath cells were increased by 3.3-fold compared to control yeasts (**Figure 7C**). This suggested that apparently increased mannan, β-glucan and total chitin may simply be due to the increased surface area of Goliath cells. Indeed microscopy revealed no obvious differences in Concanavalin A, FC-dectin or Calcofluor White fluorescence intensity between cell types (**Figure 7A**). Therefore, whilst Goliath cells may contain more chitin molecules per cell, this is unlikely to reflect an increased concentration of these molecules in the cell wall. Indeed, for mannan (1.4-fold increase) and β-glucan (2-fold increase), relative levels of these cell wall components in Goliath cell wall may be even lower than control yeasts. In contrast, chitin exposure on Goliath cells was 8-fold higher as determined by flow cytometry and when fluorescence was normalized to cell size, exposed chitin was the only component found to be higher on Goliath cells (**Figure 7B**). Moreover, fluorescence microscopy revealed an increase in the number and intensity of FITC-WGA positive puncta on the Goliath cell wall (**Figure 7A**). Therefore, Goliath cells do exhibit altered cell wall composition, associated with increased chitin exposure that occur in a non-uniform and punctate pattern across the cell wall.

### *C. albicans* Goliath Cells Show an Increased Adhesion Capacity

The capacity of *C. albicans* to adhere to both biotic and abiotic surfaces is a key pathogenicity mechanism and is also likely important for commensal colonization. However, under standard laboratory growth conditions, yeast cells adhere poorly compared to hyphae (Wachtler et al., 2011). As Goliath cells exhibited cell wall alterations (**Figure 7**) and have an increased surface area compared to control yeasts, we reasoned that Goliath cells may exhibit altered adhesive properties.

**FIGURE 8:**
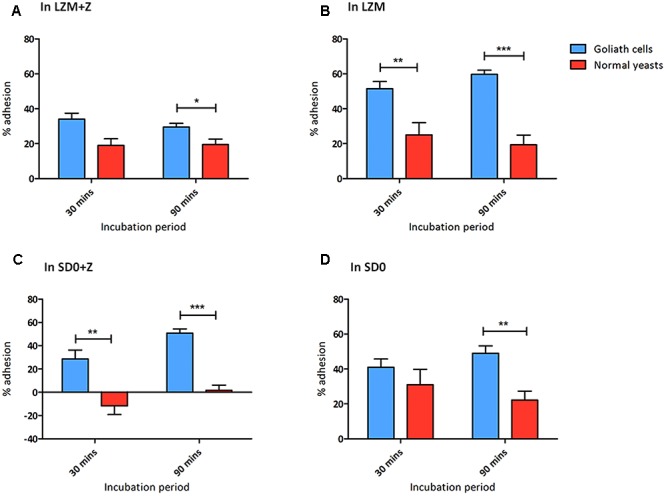
Goliath cells exhibit increased adhesion to plastic surfaces. Adherence of *C. albicans* Goliath cells formed in LZM **(A,B)** or in SD0 **(C,D)** to plastic in the presence **(A,C)** or absence **(B,D)** of zinc in the adhesion assay medium. Relevant control yeast cells formed in LZM + Z or SD0 + Z. Following incubation for 30 or 90 min, wells were washed three times with ultrapure water. CFUs in the initial supernatant and in all three wash steps were determined to calculate % adhesion. Error bars represent standard deviation and *p*-values were determined by *t*-test. Data are from three independent experiments **(A,B)**; and two independent experiments **(C,D)**. Goliath cells are more adherent than normal yeast cells in all the tested media type at both the time points. The adhesion capacity of Goliath cells is reduced upon incubation in LZM + Z (**A** vs. **B**). However, the presence of zinc during adhesion had a less pronounced effect on Goliath cell adhesion in SD0 + Z (**C** vs. **D**). Normal yeast cells show increased adhesion when incubated in the absence of zinc (**D** vs. **C**) (^∗^*P* ≤ 0.05, ^∗∗^*P* ≤ 0.01, ^∗∗∗^*P* ≤ 0.0001).

We tested the capacity of both Goliath cells (LZM preculture) and control yeasts (LZM + Z preculture) to adhere to polystyrene surfaces in the presence of either LZM or in LZM + Z (**Figures 8A,B**).

When assayed in either LZM + Z or LZM, Goliath cells adhered significantly better than control yeasts and this effect was far more pronounced when zinc was omitted from the assay medium (LZM). In fact, in LZM, Goliath cells reached 51.5 and 59.5% adhesion by 30 and 90 min, respectively.

We considered even 20% adhesion of control yeast cells in these assays surprising. In our experience *C. albicans* yeast cells do not adhere until they form hyphal germ tubes (Wilson and Hube, 2010; Wachtler et al., 2012) and in the current assay, all cells remained as either Goliath or yeast cells. The zinc-depletion medium used here (LZM/LZM + Z) contains 1 mM EDTA and only 0.2% glucose, which may already be stressful for *C. albicans*, even with zinc supplementation. It may be that LZM + Z control yeast cells themselves are already somewhat nutritionally stressed and it has previously been shown that altered carbon source, for example, can affect adhesion (Ene et al., 2012).

We therefore repeated the adhesion experiment using SD0 as the basal medium. When assayed for adhesion in SD0 + Z, control yeast cells did not adhere at all. In contrast, Goliath cells exhibited robust adhesion –41 and 50.5% by 30 and 90 min, respectively (**Figure 8C**). Interestingly, when we performed the adhesion assay itself in the absence of zinc (SD0), both Goliath and control yeast cells exhibited robust adhesion with Goliath cells being almost twice as adherent as control yeasts after 90 min.

In summary, Goliath cells are highly adherent to a polystyrene surface and, in SD0 basal medium, adhesion of control yeast cells is stimulated by short term exposure to zinc depletion (**Figures 8C,D**). Therefore both cellular and environmental zinc levels can significantly influence *C. albicans* adhesion.

### Impact of Pra1 on the Goliath Phenotype

**FIGURE 9:**
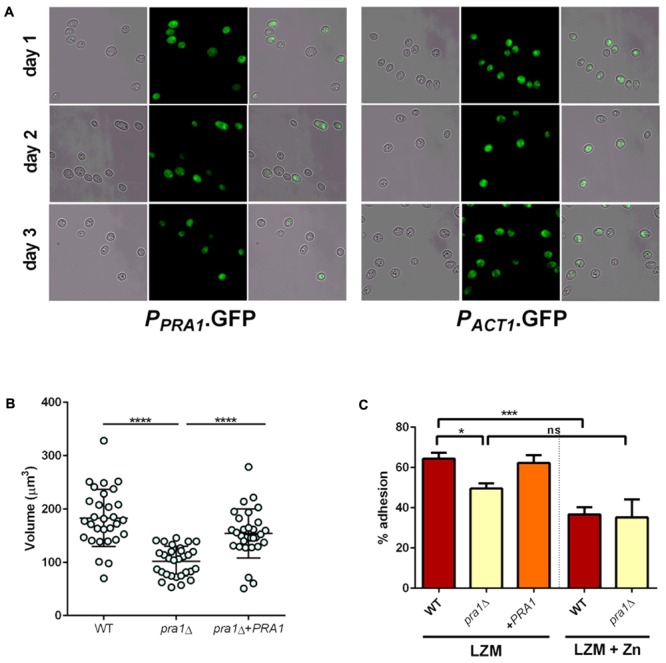
Pra1 contributes to enlargement and adhesion properties of Goliath cells. **(A)** *PRA1* is expressed in limited zinc medium (LZM). Cells harboring *P_PRA1_*-GFP (Citiulo et al., 2012) or *P_ACT1_*-GFP were analyzed at indicated time points. **(B)** *C. albicans* cells were cultured for 2 days in LZM and cell volumes were calculated by *V* = 4/3 π *ab*^2^. Data in each column represent at least 30 independent measurements from three independent experiments. *pra1*Δ cells were significantly smaller than wild type and *pra1*Δ+*PRA1* cells (*P* < 0.0001, one-way ANOVA). **(C)** Adherence of indicated *C. albicans* cells cultured for 2 days in LZM to induce cellular enlargement or for 1 day in LZM + Z to generate yeast cells. Wild type, but not *pra1*Δ exhibited enhanced adhesion following incubation in LZM (^∗^*P* < 0.05, ^∗∗^*P* < 0.01, ^∗∗∗^*P* < 0.001, ^∗∗∗∗^ *P* < 0.0001).

We have previously demonstrated a role for Pra1 in fungal adaption to zinc restriction at neutral pH (Citiulo et al., 2012; Wilson, 2015). However, the zinc limited media used in this study is acidic (∼pH 4.6–4.8) and, although responsive to zinc (Citiulo et al., 2012), it was unclear whether *PRA1* would be expressed in acidic environments (Sentandreu et al., 1998; Bensen et al., 2004). We therefore incubated cells harboring either empty vector (negative control), *P_PRA*1*_*-GFP, or *P_ACT*1*_*-GFP (positive control) in LZM and assessed GFP expression. Interestingly, at each time point analyzed, the majority of the *P_PRA*1*_*-GFP cell population expressed GFP at levels similar to the *P_ACT*1*_*-GFP control (**Figure 9A**). Therefore, in addition to being a pH-regulated antigen, *PRA1* can also be expressed at acidic pH when environmental zinc highly restricted.

Having established expression, we next tested whether deletion of *PRA1* affected Goliath cell formation itself. Isogenic wild type, *pra1*Δ and *pra1*Δ+*PRA1* cells were incubated and cell size determined as before. Although, *pra1*Δ cells did enlarge upon zinc restriction (reaching a mean cell volume of 102 μm^3^), these cells were significantly smaller than wild type Goliath cells (183 μm^3^) and complementation with a single copy of *PRA1* significantly restored cell size to 154 μm^3^ (**Figure 9B**). Therefore, although not essential for cellular enlargement in response to zinc restriction (the volume of regular yeast cells is approximately 30 μm^3^), Pra1 does positively contribute to Goliath cell formation.

Given the observed defect in enlargement of *pra1*Δ, we next tested the impact of this on adhesion. Cells were cultured in LZM to induce enlargement and adhesion again assessed in the presence of LZM. Interestingly, *PRA1* copy number strongly correlated with both cell size and adhesion, with wild type (*PRA1*/*PRA1*), *pra1*Δ and *pra1*Δ+*PRA1* strains exhibiting 64, 49.6, and 62.3%, respectively (**Figure 9C**). In fact, the *R*^2^-value of the correlation between cell volume and adhesion potential was 0.948 (based on a linear equation of normal yeast, and enlarged cells of *pra1*Δ, *pra1*Δ+*PRA1*, wild type adhesion over volume). These data indicate that the size of Goliath cells themselves contributes to their increased adhesion.

### The Effect of Environmental pH and Temperature on Goliath Cell Formation

**FIGURE 10:**
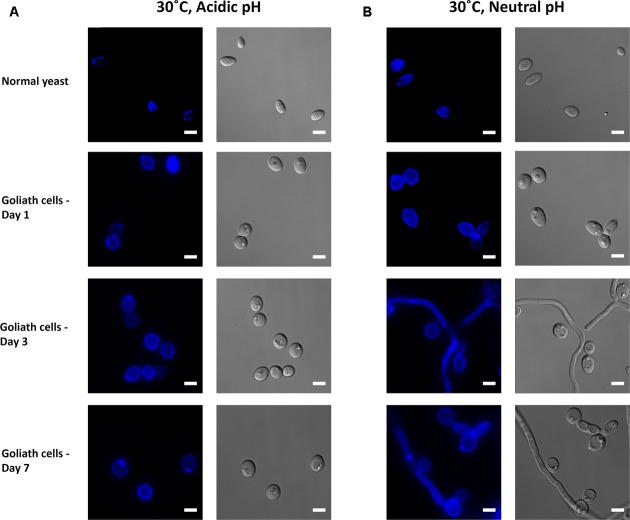
Impact of pH on Goliath morphology. *C. albicans* cells were incubated in limited zinc media (LZM) at 30°C at acidic (pH 4.6) or neutral-alkaline (pH 7.3) and morphology assessed. **(A)** In acidic medium zinc limitation induces cellular enlargement. **(B)** At pH 7.3 zinc limitation induces yeast cell enlargement and triggers filamentation. Experiment was performed twice.

*Candida albicans* morphogenesis is exquisitely sensitive to the cells’ ambient environment. In particular, temperature and pH are important morphogenic cues: low temperature and acidic pH promote the yeast morphology; whilst physiological temperature (37°C) and neutral-to mildly alkaline pH promote the hyphal morphology. We therefore tested the effect of zinc-, pH-, and temperature on *C. albicans* morphology. At 30°C, alkalinizing the medium to pH 7.3 had no obvious impact on *C. albicans* yeast morphology in zinc replete conditions (**Figures 10A,B**). In contrast, in the absence of zinc, cells initially enlarged, and then began to filament (**Figure 10B**). At these later time points, very little filamentation was observed in the presence of zinc (not shown).

**FIGURE 11:**
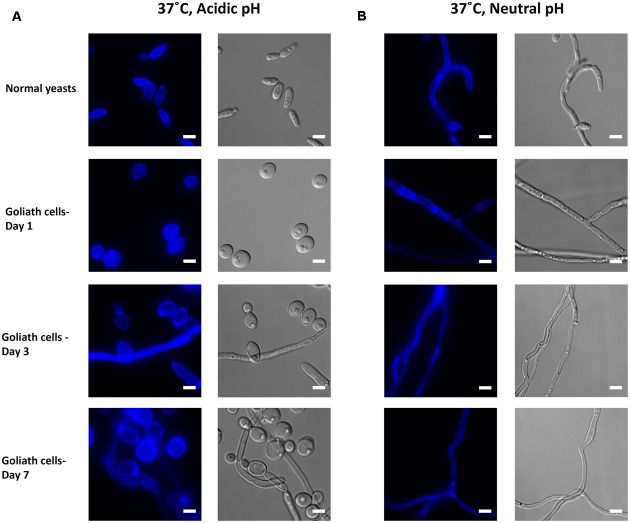
Impact of temperature on Goliath morphology. *C. albicans* cells were incubated in limited zinc media (LZM) at 37°C at acidic (pH 4.6) or neutral-alkaline (pH 7.3) and morphology assessed. **(A)** In acidic medium at 37°C zinc limitation induces a greater degree of cellular enlargement than at 30°C (compare **Figure 10A**) and pseudohypha development. **(B)** At pH 7.3 and 37°C filamentation occurs independent of media zinc status. Experiment was performed twice.

In zinc replete acidic medium at 37°C, *C. albicans* cells grew as slightly elongated yeasts (**Figure 11A**). Under zinc limitation, Goliath cell formation was even more pronounced at 37°C than at 30°C (compare **Figures 10A**, **11A**) and some filamentation was observed, however, invaginations at the septa indicated that these cells were mainly pseudohyphae. Therefore physiological temperature (37°C) stimulates Goliath cell formation. Finally in neutral medium at 37°C, all cells formed hyphae independent of zinc status (**Figure 11B**). This was unsurprising as pH 7.3, together with 37°C is a strong inducer of hypha formation (Sudbery, 2011).

In summary environmental zinc restriction is a novel trigger for *C. albicans* adhesion, associated with a notable increase in cell size.

## Discussion

*Candida albicans* polymorphism is one of the most widely recognized attributes of this important human fungal pathogen (Lu et al., 2014; Jacobsen and Hube, 2017; Noble et al., 2017). The most well studied morphologies are yeast and hyphae and the yeast to hypha dimorphic transition is thought to be a key virulence factor. This is because multiple virulence-associated genes which encode factors required for adhesion (e.g., Hwp1, Als3), invasion (Als3) and damage (Ece1) are hardwired into the hyphal morphogenetic program (Martin et al., 2013). Whilst hyphae are considered the dominant invasive morphology, yeast cells are thought to be important for commensal colonization, dissemination in the bloodstream and reseeding from biofilms during recurrent candidaemia (Kullberg and Arendrup, 2015). The role of other morphologies in commensalism and disease, including pseudohyphae and chlamydospores, are not as well understood. In addition to these morphological transitions, yeast cells can also undergo phenotypic switching. The most well studied of these is the white-opaque switch, which is important for mating in *C. albicans* (Hickman et al., 2013). More recently, Pande et al. (2013) described a novel yeast phenotype. The authors found that gastrointestinal passage of a strain over-expressing the transcription factor Wor1 resulted in a switch to an elongated yeast cell morphology. These GUT cells (gastrointestinal induced transition), which were distinct from the opaque phenotype, had increased gastrointestinal (GI) tract fitness and differentially regulated genes involved in iron acquisition, glucose metabolism and phosphate uptake.

Here we describe and characterize another *C. albicans* phenotype: the Goliath cell. This form of cellular enlargement occurred in response to zinc limitation, but not to iron-, manganese- or copper- restriction. The group of Soll has also observed *C. albicans* cellular enlargement in response to zinc limitation (Bedell and Soll, 1979) and this, combined with our own observations that all *C. albicans* clinical isolates tested underwent this transition, strongly suggests that this is a conserved morphological response in *C. albicans*. Enlarged yeast cells have been observed under other laboratory conditions such as during stationary phase growth of a genetic mutant lacking *ECM33* (Gil-Bona et al., 2016), on IsoVitalex-supplemented chocolate agar (Bottone et al., 1999) or in response to xylitol (Ameglio et al., 1990). Importantly, enlarged yeast cells have also been observed from a patient suffering from systemic candidiasis (Alasio et al., 2003).

Zinc limitation is relevant to both *Candida* commensal colonization and pathogenicity. In the GI tract, active inflammation results in high levels of calprotectin in the gastrointestinal lumen. In fact, calprotectin is amongst the best studied markers of gastrointestinal inflammation (Walsham and Sherwood, 2016). Calprotectin is a heterodimeric protein composed of S100A8 and A9 subunits and has potent antimicrobial activity (Urban et al., 2009). Its antifungal activity is mediated by zinc chelation and calprotectin has high affinity for this metal, with a dissociation constant in the low nanomolar range (Kehl-Fie et al., 2011). However, *C. albicans* also produces a zinc binding (“zincophore”) protein, encoded by *PRA1* (Citiulo et al., 2012). Like host calprotectin, Pra1 has low nanomolar affinity for zinc (Loboda and Rowinska-Zyrek, 2017), and this zincophore allows the fungus to scavenge for zinc from host tissue. Here we demonstrate a role for Pra1 in Goliath cell enlargement and adhesive potential. Therefore, in the context of its evolutionary history as GI tract commensal, *C. albicans* likely experiences zinc restriction during gastrointestinal inflammation. Superficial mucosal infections, such as vulvovaginal candidiasis, can also be associated with calprotectin and neutrophil influx (Yano et al., 2012). Moreover, when *C. albicans* enters normally sterile site, such as the bloodstream, it will likely face extreme zinc limitation due to the coordinate action of nutritional immunity (Crawford and Wilson, 2015).

Beyond their size, we observed three major Goliath cell phenotypes: (i) hyper-adherence, (ii) delayed hyphal morphogenesis and (iii) increased chitin exposure. (i) The high adhesive potential of Goliath cells was unexpected. In our experience (Wilson and Hube, 2010; Wachtler et al., 2011), *C. albicans* yeast cells adhere very poorly to both biotic and abiotic surfaces under laboratory conditions. At this stage, the molecular mechanism of zinc depletion-induced adhesion is unclear, but may involve Zap1. This transcription factor is responsive to changes in zinc availability and is the main regulator of zinc homeostasis in fungi (Ballou and Wilson, 2016; Wilson and Bird, 2016). Interestingly, as well as regulating genes involved in zinc assimilation and homeostasis, Zap1 in *C. albicans* also controls the expression of the Cell surface targets of adherence regulators (CSTAR) genes, a regulon composed of multiple putative and described adhesin genes (Nobile et al., 2009; Finkel et al., 2012). It is therefore likely that the increased adhesion phenotype of Goliath cells is the result of zinc-depletion activation of Zap1. Indeed, we observed that deletion of *PRA1* (a major Zap1 target) inhibited both cellular enlargement and adhesion. Although Pra1 has been implicated in the binding of *C. albicans* to host cells and various immune mediators (Soloviev et al., 2007; Zipfel et al., 2011), we think it is unlikely that Pra1 is directly functioning as an adhesin on *C. albicans* Goliath cells. It is possible that impaired zinc homeostasis in the *pra1*Δ mutant inhibits cellular enlargement, and that it is simply this enlargement defect which results in reduced adhesion of the *pra1*Δ mutant. Indeed, the observed correlation between cell size and adhesive potential indicates that the larger surface area of Goliath cells may contribute to adhesion by providing a larger point of contact between the fungus and the substratum.

(ii) The hyphae-inducing medium used in this study (RPMI, 37°C, 5% CO_2_) is a potent inducer of hyphal morphogenesis, and yet Goliath cells exhibited a lengthy delay before the initiation of germ tube formation in this medium. Therefore, compared to normal yeast cells, Goliath cells appear to be less primed to form hyphae. In natural environments, *C. albicans* faces more complex environments, and its morphological output will depend on the integration of these multiple stimuli (Sudbery, 2011). As hypha formation may represent a commitment to invasive growth (Mech et al., 2014), and is intimately associated with host cell damage (Moyes et al., 2016), reduced hypha formation on host surfaces may be associated with reduced damage and immune activation. On the other hand, under conditions of elevated pH and low temperature, zinc restriction promoted filamentation. It would therefore appear that environmental zinc is set to take its place amongst the plethora of environmental cues which influence morphogenesis in *C. albicans* (Sudbery, 2011).

(iii) Finally, of the cell wall components tested in this study, only chitin exposure was increased relative to cell size on the Goliath cell wall. This observation may have implications for immune interactions (Wagener et al., 2014). As well as providing structural stability to the cell wall (Gow and Hube, 2012; Gow et al., 2017), chitin can also be recognized by the host immune system. Depending on the size of chitin biomolecule, it has been shown to trigger either a pro-inflammatory or an anti-inflammatory response. Mora-Montes et al. (2011) showed that ultrapure chitin from the cell wall of *C. albicans* failed to elicit a cytokine response in human peripheral blood monocytes (PBMCs) and that PBMCs pre-incubated with ultrapure chitin did not readily recognize live *C. albicans* cells, causing a significant reduction in cytokine production.

Together, these three traits may facilitate a non-damaging “persister” phenotype: the capacity of *C. albicans* Goliath cells to colonize host surfaces via adhesion, whist presenting increased chitin and greatly diminished hypha formation may allow the cells to avoid triggering damage and inflammation. We are now investigating the impact of this morphotype on host pathogen interactions and immune evasion.

Intriguingly, from an evolutionary perspective, cellular enlargement in response to zinc limitation was observed for *C. albicans*, *C. dubliniensis*, and *C. tropicalis*: the three most closely related commonly studied *Candida* species (Butler et al., 2009). Although all three species are common pathogens of humans, it is unlikely that this cellular response originated within the context of colonization of humans. The age of the CUG-Ser clade (to which these three species belong) has been estimated at 170 million years (Massey et al., 2003). The two more closely related species, *C. albicans* and *C. dubliniensis* appear to have diverged 20 million years ago (McManus and Coleman, 2014). It is unclear when the more distantly related *C. tropicalis* diverged from their common ancestor: based on these divergence estimates and branch length reported in Butler et al. (2009), this may have occurred between approximately 57 and 70 million years ago. Therefore, it would appear that cellular enlargement in response to zinc limitation has been maintained for tens of millions of years and remains a morphological property of at least three medically important extant species.

## Materials and Methods

### Strains and Growth Conditions

Strains (*C. albicans* clinical isolates and other *Candida* species) used in this study are listed in Supplementary Table S1. The isogenic wild type *C. albicans* strain (BWP17+CIp30) was used for most experiments.

All strains were maintained on YPD agar (1% yeast extract 2% mycological peptone, 2% D-glucose and 2% agar). Liquid overnight cultures were grown in SD (2% glucose, 2% YNB, 0.5% NH_4_SO_4_) medium in a shaking incubator at 30°C and 200 rpm.

### Metal Limitation

To elicit acute zinc limitation, cells were precultured in SD media, washed three times with ultrapure water and inoculated at an OD_600_ 0.2 in 4 ml LZM (media composition listed in Supplementary Table S2) in a universal tube. Tubes were incubated at 30°C, 200 rpm for 3 days. As an alternative method of zinc starvation, SD pre-grown cells were washed and inoculated at an OD_600_ 0.05 in 4 ml SD0 (2% glucose without zinc, 2% YNB without zinc, Formedium). Tubes were incubated similarly for 7 days. Control cultures were supplemented with 25 μM ZnSO_4_ (LZM + Z/SD0 + Z) and incubated for 1 day.

To monitor increases in cell size, *C. albicans* (WT) Goliath cells (LZM/SD0) and normal yeasts (LZM + Z/SD0 + Z) were imaged and optical densities were measured at OD_600_. Imaged cells were analyzed using ImageJ v1.47and cell volume determined using the formula V = 4/3 π *ab*^2^ where *a* is the radius of the major axis and *b* is the radius of the minor axis of the cell.

The effect of zinc limitation was tested on *C. albicans* clinical isolates and *Candida* species listed in Supplementary Table S1 by incubating SD precultured cells in LZM and/or LZM + Z as described above. Following incubation, cells were imaged using Deltavision-RT at 100× magnification.

To induce iron, copper and manganese limitation, WT *C. albicans* cells precultured in SD media were washed and inoculated at an OD_600_ 0.2 in 4 ml LIM, LCM, and LMM (media composition in Supplementary Table S2) in a universal tube. Tubes were incubated at 30°C, 200 rpm for 3 days. Control cultures were supplemented with FeSO4, CuSO4, and MnSO4, respectively, as described in Supplementary Table S2 and incubated for 1 day.

### Hypha Induction

To assess the capacity of Goliath cells to form hyphae, *C. albicans* WT cells cultured for 3 days in LZM and normal yeasts cultured in LZM + Z for 1 day were washed three times with ultrapure water and 10^5^ cells were inoculated in RPMI 1640 (Gibco) in a 12 well plate. Plates were incubated at 37°C and 5% CO_2_ for 90, 180, or 360 min, fixed and stained with stained with Calcofluor White. Cells were imaged using DeltaVision at 40× magnification.

### Cell Wall Staining

To assess the effect of *C. albicans* Goliath cells exposure to zinc, WT cells incubated for 2 days in LZM were washed 3× with ultrapure water and stained with 10 μg/ml FITC in carbonate-bicarbonate buffer (pH 9.6) for 20 min in the dark at room temperature. Stained cells were washed with ultrapure water and inoculated in SD media at an OD_600_ 0.2 in a universal tube. Tubes were incubated for 16 h as previously described. Following incubation cells were washed and fixed in Histofix 4% (Roth^®^) for 45 min at 30°C. Fixed cells were washed and imaged using FITC filter channel in Deltavision-RT at 100× magnification.

To determine potential differences in cell wall composition, 2.5 × 10^6^ Goliath cells (cultured in LZM for 3 days) and normal yeasts (grown for 1 day in LZM + Z) were fixed and washed three times with ultrapure water. Fixed cells were stained independently with 25 μg/ml Calcofluor White (CFW), 100 μg/ml wheat germ agglutinin (WGA), 50 μg/ml concanavalin A (ConA) or 5 μg/ml FC-Dectin. Cells were incubated for 45 min to 1 h at room temperature in the dark. Following incubation, cells were washed twice with PBS containing 1% fetal calf serum and 0.5 mM EDTA. Stained cells were analyzed using flow cytometry and fluorescence microscopy. For imaging, cells were analyzed using appropriate filter channels (DAPI, Rh-TRITC, and FITC) in DeltaVision-RT at 100× magnification. Flow cytometry data was obtained using BD Fortessa and mean fluorescence intensities were measured using Alexa 350 and Ds-Red channels. Forward and side scatter were measured to determine difference in cell size.

### Adhesion Assay

*Candida albicans* WT cells (10^5^ cells/ml) pre-grown in LZM for 2 days and LZM + Z for 1 day were further incubated in LZM and LZM + Z in a 12 well Falcon^TM^ plate at 30°C for 0, 30, and 90 min. Following incubation, supernatant from wells was collected and wells were washed three times with sterile ultrapure water. Washes from each well were also collected. Serial dilutions were prepared for supernatant and washes that were then plated on to YPD agar. CFUs obtained following incubation were counted to determine the percentage of detached and adherent cells at each time-points. This protocol was repeated with WT *C. albicans* cells pre-grown in SD0 for 7 days and SD0 + Z for 1 day.

### Statistical Analysis

All data were obtained from at least two independent experiments. At least 100 cells (and 35 cells for day 0) were measured for determination of cell size over three independent experiments. Statistical analysis was performed on GraphPad Prism5 using Students *t*-test and ANOVA as appropriate.

## Author Contributions

DW, BH, and NG conceived the study. DM, LL-M, OA, EW, and DW acquired, analyzed, and interpreted the data. DM and DW drafted the manuscript. LL-M, OA, EW, NG, and BH critically revised the manuscript. All authors approved the final submitted version and agree to accountability for all aspects of the work.

## Conflict of Interest Statement

The authors declare that the research was conducted in the absence of any commercial or financial relationships that could be construed as a potential conflict of interest.
