# Supplementary material for: Zinc Limitation Induces a Hyper-Adherent Goliath Phenotype in Candida albicans
Source: Front Microbiol. 2017 Nov 14;8:2238. doi: 10.3389/fmicb.2017.02238 (PMC5694484; doi:10.3389/fmicb.2017.02238)
Supplement: Supplementary file 1 [file Table_1.DOCX]

Table S1 : *C. albicans* strains and clinical isolates and *Candida* species used in this study .

| **Strain** | **Species** | **Source** | **Reference** |
| --- | --- | --- | --- |
| BWP17 + Clp 30 (Wild type) | *Candida albicans* | BWP17 | (Mayer et al, 2012; Wilson et al, 1999) |
| *pra1*Δ | *Candida albicans* | BWP17 | (Citiulo et al, 2012) |
| *pra1*Δ+*PRA1* | *Candida albicans* | BWP17 | (Citiulo et al, 2012) |
| RIH09 | *Candida albicans* | Blood | (MacCallum et al, 2009) |
| HUN96 | *Candida albicans* | Blood | (MacCallum et al, 2009) |
| IHEM16614 | *Candida albicans* | Oropharynx | (MacCallum et al, 2009) |
| AM2003/0182 | *Candida albicans* | Blood | (MacCallum et al, 2009) |
| J990102 | *Candida albicans* | Vagina | (MacCallum et al, 2009) |
| FC28 | *Candida albicans* | Vagina | (MacCallum et al, 2009) |
| LI086 | *Candida albicans* | Wound | (MacCallum et al, 2009) |
| S20176.079 | *Candida albicans* | Blood | (MacCallum et al, 2009) |
| Wü284 | *Candida dubliniensis* | Clinical (unknown) | (Morschhauser et al, 1999) |
| CD36 | *Candida dubliniensis* | Oral | (Sullivan et al, 1995) |
| MYA-3404 | *Candida tropicalis* | Reference strain | (Butler et al, 2009) |
| B31581/7/04 | *Candida tropicalis* | Urine | (Walker et al, 2013) |
| SCS40113 | *Candida parapsilosis* | Blood | (Walker et al, 2013) |
| ATCC 22019 | *Candida parapsilosis* | Reference strain | (Guerin et al, 1989) |
| ATCC 42720 | *Candida lusitaniae* | Reference strain | (Butler et al, 2009) |
| J960088 | *Candida famata* | Human skin lesions | Strain collection of Dr. Donna MacCallum, MRC Centre for Medical  Mycology at the University of Aberdeen |
| J960089 | *Candida famata* | Human skin lesions |  |

Table S2. Limited zinc media (LZM).

| **Stock** | **Fold conc.** | **Component** | **Stock conc. (M)** | **Final conc. (M)** | **Vol.** |
| --- | --- | --- | --- | --- | --- |
| 1 | 500 | Na_2_EDTA.2H_2_O | 5.0x10^-1^ | 1.0x10^-3^ | 1ml |
| 2 | 100 | MgSO_4_.7H_2_O  NaCl | 5.0x10^-1^  1.0x10^-1^ | 5.0x10^-3^  1.0x10^-3^ | 5ml |
| 3 | 100 | CaCl_2_.2H_2_O | 1.0x10^-1^ | 1.0x10^-3^ | 5ml |
| 4 | 100 | uridine  L-histidine  L-leucine  L-lysine | 4.0x10^-2^  5.0x10^-2^  7.6x10^-2^  7.0x10^-2^ | 4.0x10^-4^  5.0x10^-4^  7.6x10^-4^  7.0x10^-4^ | 5ml |
| 5 | 100 | (NH_4_)_2_SO_4_ | 3.8 | 3.8x10^-2^ | 5ml |
| 6 | 100 | KH_2_PO_4_ | 1.0x10^-1^ | 1.0x10^-3^ | 5ml |
| 7 | 50 | Na_3_citrate.2H_2_O | 1.0 | 2.0x10^-2^ | 10ml |
| 8 | 20 | D-glucose | 2.2x10^-1^ | 1.1x10^-2^ | 25ml |
| 9 | 1000 | d-biotin  Ca pantothenate  myo-inositol  pyridoxin  thiamin.HCl | 1.6x10^-5^  1.7x10^-3^  1.0x10^-2^  2.0x10^-3^  1.0x10^-3^ | 1.6x10^-8^  1.7x10^-6^  1.0x10^-5^  2.0x10^-6^  1.0x10^-6^ | 0.5ml |
| 10 | 10000 | H_3_BO_3_  KI  Na_2_MoO_4_.2H_2_O | 1.0x10^-1^  5.0x10^-3^  1.0x10^-2^ | 1.0x10^-5^  5.0x10^-7^  1.0x10^-6^ | 50µl |

The pH values of stocks 1 and 7 were adjusted to 8.0 and 4.2, respectively, and Stock 10 was prepared in 0.1 M HCl. Solutions were filter-sterilized with 0.2 µm cellulose nitrate filters (Schleicher & Schuell) and stored in polycarbonate bottles. To prepare the different LXM media, the stock solutions were sequentially added to ultra-pure water, filter-sterilized and stored in polycarbonate bottles. To generate the respective metal limited media, the following transition metals were added: FeCl (6.17 µM), MnSO_4_ (13.24 µM), CuSO_4_ (0.3 µM) and ZnSO_4_ (25 µM). To generate LZM pH 7.3, media was alkalinised with NaOH and buffered with 50 mM HEPES pH 7.4

Butler G, Rasmussen MD, Lin MF, Santos MAS, Sakthikumar S, Munro CA, Rheinbay E, Grabherr M, Forche A, Reedy JL, Agrafioti I, Arnaud MB, Bates S, Brown AJP, Brunke S, Costanzo MC, Fitzpatrick DA, de Groot PWJ, Harris D, Hoyer LL, Hube B, Klis FM, Kodira C, Lennard N, Logue ME, Martin R, Neiman AM, Nikolaou E, Quail MA, Quinn J, Santos MC, Schmitzberger FF, Sherlock G, Shah P, Silverstein KAT, Skrzypek MS, Soll D, Staggs R, Stansfield I, Stumpf MPH, Sudbery PE, Srikantha T, Zeng QD, Berman J, Berriman M, Heitman J, Gow NAR, Lorenz MC, Birren BW, Kellis M, Cuomo CA (2009) Evolution of pathogenicity and sexual reproduction in eight Candida genomes. *Nature* **459:** 657-662

Citiulo F, Jacobsen ID, Miramon P, Schild L, Brunke S, Zipfel P, Brock M, Hube B, Wilson D (2012) *Candida albicans* scavenges host zinc via Pra1 during endothelial invasion. *PLoS pathogens* **8:** e1002777

Guerin M, Camougrand N, Caubet R, Zniber S, Velours G, Manon S, Guelin E, Cheyrou A (1989) The second respiratory chain of Candida parapsilosis: a comprehensive study. *Biochimie* **71:** 887-902

MacCallum DM, Castillo L, Nather K, Munro CA, Brown AJP, Gow NAR, Odds FC (2009) Property Differences among the Four Major Candida albicans Strain Clades. *Eukaryot Cell* **8:** 373-387

Mayer FL, Wilson D, Jacobsen ID, Miramon P, Grosse K, Hube B (2012) The novel *Candida albicans* transporter Dur31 Is a multi-stage pathogenicity factor. *PLoS pathogens* **8:** e1002592

Morschhauser J, Ruhnke M, Michel S, Hacker J (1999) Identification of CARE-2-negative Candida albicans isolates as Candida dubliniensis. *Mycoses* **42:** 29-32

Sullivan DJ, Westerneng TJ, Haynes KA, Bennett DE, Coleman DC (1995) *Candida dubliniensis* sp. nov.: phenotypic and molecular characterization of a novel species associated with oral candidosis in HIV-infected individuals. *Microbiology* **141 ( Pt 7):** 1507-1521

Walker LA, Gow NA, Munro CA (2013) Elevated chitin content reduces the susceptibility of Candida species to caspofungin. *Antimicrob Agents Chemother* **57:** 146-154

Wilson RB, Davis D, Mitchell AP (1999) Rapid hypothesis testing with *Candida albicans* through gene disruption with short homology regions. *Journal of bacteriology* **181:** 1868-1874
